# Antioxidant Activity of Some Plant Extracts Towards Xanthine Oxidase, Lipoxygenase and Tyrosinase

**DOI:** 10.3390/molecules14082947

**Published:** 2009-08-10

**Authors:** Chin-Hui Chen, Hsiu-Chen Chan, Yi-Tsu Chu, Hsin-Yi Ho, Pi-Yu Chen, Tzong-Huei Lee, Ching-Kuo Lee

**Affiliations:** 1 College of Pharmacy, Taipei Medical University, 250 Wu Xin Street, Taipei 11031, Taiwan; E-mails: chinhui@mail.ypu.edu.tw (C-H.C.), dorashuu@hotmail.com (H-C.C.), liopayi@msn.com (Y-T.C.), bleaktruth76@hotmail.com (H-Y.H.), thlee@tmu.edu.tw (T-H.L.), e023089103@tmu.edu.tw (P-Y.C); 2 Department of Medical Laboratory Science and Biotechnology, Yuanpei University, 306 Yuanpei St, Hsinchu 300, Taiwan; E-mail: chinhui@mail.ypu.edu.tw (C-H.C.)

**Keywords:** HPLC-DAD, antioxidant, plant extracts, xanthine oxidase, tyrosinase, lipoxygenase

## Abstract

Natural products have the potential to be developed into new drugs for the treatment of various diseases. The aim of the present study was to screen the antioxidant activities of some common edible fruits, garden plants and medicinal plants indigenous to Taiwan. This was performed by assessing the activities of lipoxygenase, xanthine oxidase and tyrosinase following incubation with extracts from these plants. A further aim was to use HPLC-DAD and tyrosinase to chromatographically identify the antioxidative constituents obtained from an extract exhibiting strong antioxidative properties. The acetone extracts of 27 cultivated plant species from Taiwan were tested for antioxidant activities towards xanthine oxidase, tyrosinase and lipoxygenase using spectrophotometric assays. *Koelreuteria henryi*, *Prunus campanulata*, and *Rhodiola rosea* showed the highest xanthine oxidase inhibitory activities. *Camellia sinensis*, *Rhodiola rosea*, and *Koelreuteria henryi* exhibited good tyrosinase inhibitory activities and potent anti-lipoxygenase activities. As *Koelreuteria henryi* had notable significant inhibitory activities towards xanthine oxidase, tyrosinase, and lipoxygenase, it was further tested with tyrosinase and HPLC-DAD. The results from this part of the study revealed that the more powerful the antioxidant capability of the extracted component, the greater the decrease in peak height obtained after reacting with tyrosinase. Additional studies are warranted to further characterize the compounds responsible for the antioxidant properties of the examined extracts.

## Introduction

The condition of *in vivo* “oxidative stress” is defined as elevated levels of free radicals or other reactive oxygen species (ROS) which can elicit either direct or indirect damage to the body. Plant extracts or secondary metabolites have served as antioxidants in phytotherapeutic medicines to protect against various diseases for centuries [1]. Natural antioxidants exhibit a wide range of pharmacological activities, and have been shown to have anticancer, anti-inflammatory and anti-aging properties [2,3,4,5]. Numerous vegetables, crops, spices and medicinal herbs have been tested in an effort to identify new and potentially useful antioxidants [6,7,8,9]. More recently, it has become evident that phenolic natural products may reduce oxidative stress by indirect antioxidant action. For example, various flavonoids, which are found naturally in fruits, vegetables and some beverages, have been demonstrated to exert antioxidant effects through a number of different mechanisms [10].

Tropical and subtropical regions of the world contain a vast source of natural products, some of which may have the potential to be developed into new drugs for treament various diseases. In these areas, ethnobotanical medicine often exists, offering a rich and relatively untapped potential source of new drugs that could be derived from natural products. Taiwan, a tropical Southeast Asian country, has a long history of traditional medicine. To date, however, there has been little rigorous scientific study of these traditional medicines and indigenous plants. The aim of the present study was to screen the antioxidant activities of common edible fruits, garden plants and medicinal plants indigenous to Taiwan. This was performed by assessing the activities of lipoxygenase (LOX), xanthine oxidase (XO) and tyrosinase following incubation with extracts from these plants. A further aim was to use HPLC-DAD and tyrosinase to chromatographically identify the antioxidative constituents obtained from an extract exhibiting strong antioxidative properties.

## Results and Discussion

Table 1 lists the 27 species belonging to 23 families that were examined for inhibitory effects on XO, tyrosinase, and LOX activity. The list encompasses fruits, herbs, flowers, shrubs and trees that are native to Taiwan.

**Table 1:** Yields of 27 acetone plant extracts.

No.	Plant	Part	Family	Voucher specimen	% yield
1	*Agave sisalana Perr. ex Enghlm.*	leaves	Agavaceae	M-69	1.2
2	*Alternanthera bettzickiana (Regel) Nicholsen*	aerial	Amaranthaceae	M-78	5.7
3	*Antrodia cinnamomea Chang & Chou*	carpophore	Polyporeceae	M-75	11.3
4	*Astragalus membranaceus Bge.*	roots	Leguminosae	M-76	6.9
5	*Calocedrus macrolepis Kurz var. formosana Folrin*	leaves	Cupressaceae	M-83	4.1
6	*Camellia sinensis (L.) O. Ktze.*	leaves	Theaceae	M-70	3.4
7	*Camptotheca acuminata Decne.*	leaves	Nyssaceae	M-74	5.6
8	*Chamaecyparis formosensis*	leaves	Cupressaceae	M-71	4.8
9	*Chamaecyparis obtusa var. formosana (Hayata) Rehder*	leaves	Cupressaceae	M-77	3.9
10	*Citrus ponki (Hayata) Hort. ex Tanaka*	peel	Rutaceae	M-73	7.7
11	*Cyclocodon lancifolia (Roxb.) Kurz subsp. lancifolia*	aerial	Campanulaceae	M-84	2.9
12	*Dendrobium officinale K. KIMURA et MIGO*	aerial	Orchidacea	M-81	2.3
13	*Euphorbia formosana Hayata*	aerial	Euphorbiaceae	M-59	4.7
14	*Helicia formosana Hemsl.*	seed	Proteaceae	M-68	10.5
15	*Garcinia subelliptica Merr.*	fruit	Clusiaceae	M-65	14.5
16	*Gynura bicolor (Willd.) DC.*	leaves	Asteraceae	M-66	8.1
17	*Koelreuteria henryi Dumme*	leaves	Sapindaceae	M-73	6.7
18	*Passiflora edulis Sims*	peel	Lauraceae	M-60	11.4
19	*Persea americana Mill*	fruit	Passifloraceae	M-64	7.8
20	*Persea americana Mill*	leaves	Passifloraceae	M-61	5.4
21	*Phyllanthus urinaria L.*	aerial	Euphorbiaceae	M-67	3.3
22	*Pittosporum tobira*	leaves	Pittosporaceae	M-63	4.6
23	*Prunus campanulata Maxim*	leaves	Rosaceae	M-94	4.2
24	*Rhodiola rosea L* *.*	roots	Crassulaceae	M-91	9.0
25	*Ruellia tuberosa L.*	aerial	Acanthaceae	M-48	7.9
26	*Syzygium samarangense (Blume) Merr. & Perry*	fruit	Myrtaceae	M-55	1.3
27	*Washingtonia filifera (Linden ex Andre) Wendl.*	leaves	Arecaceae	M-75	2.9

### Xanthine oxidase

**Figure 1:**
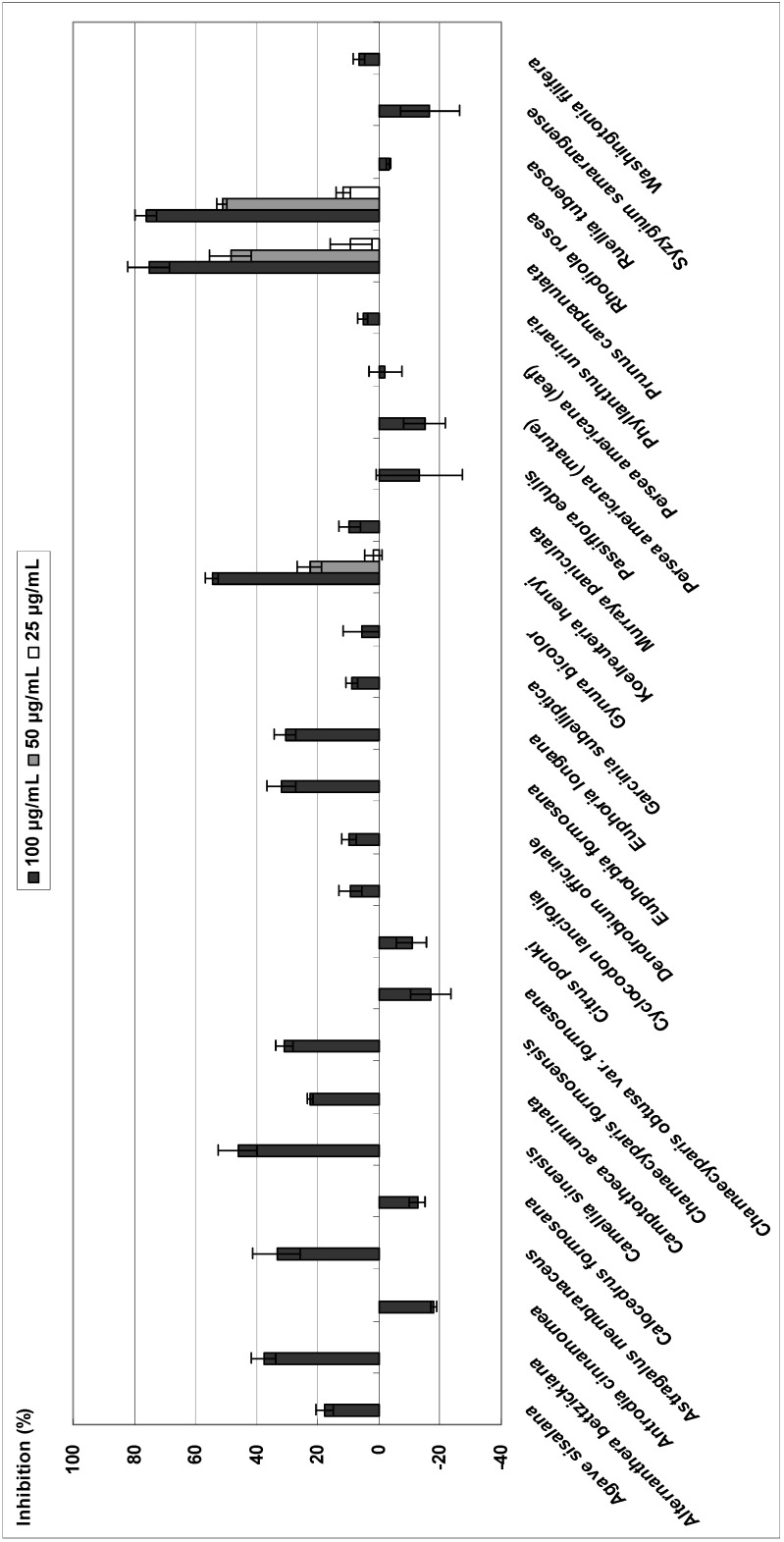
Inhibitory effect of 27 plant acetone extracts on xanthine oxidase activity.

The 27 plant extracts were screened at a concentration of 0.1 mg/mL. The extracts exhibiting greater than 50% of XO inhibitory activity were further investigated to determine the concentration that could inhibit 50% of enzyme activity (IC_50_). At 0.1 mg/mL, three plant extracts (*Koelreuteria henryi*, *Prunus campanulata*, and *Rhodiola rosea*) exhibited >50% inhibition of XO activity. Inhibition of XO activity by the remaining extracts was below 50% or not inhibitory. The results are summarized in Figure 1. In total, the crude extracts possessing XO inhibitory activity with IC_50_ values less than 100 μg/mL were acetone extracts of *Koelreuteria henryi* (IC_50_, 91.8 ± 1.7 μg/mL), *Prunus campanulata* (IC_50_, 64.6 ± 5.8 μg/mL) and *Rhodiola rosea* (IC_50_, 56.0 0 ± 1.0 μg/mL). The IC_50_ concentration for quercetin was 1.1 ± 0.5 μg/mL (3.6 μM). The XO inhibitory activities of *Koelreuteria henryi* and *Prunus campanulata* have not previously been reported. Past studies have demonstrated that *Rhodiola rosea* exerts inhibitory activity against XO [11]. Screening revealed that three plant extracts showed higher XO inhibitory effects than quercetin. Further study is warranted to confirm that the extracts from these plant extracts inhibit XO activity *in vivo*. It would also be interesting to further isolate and elucidate the chemical structures of the XO inhibiting compounds in these extracts.

### Tyrosinase

**Figure 2:**
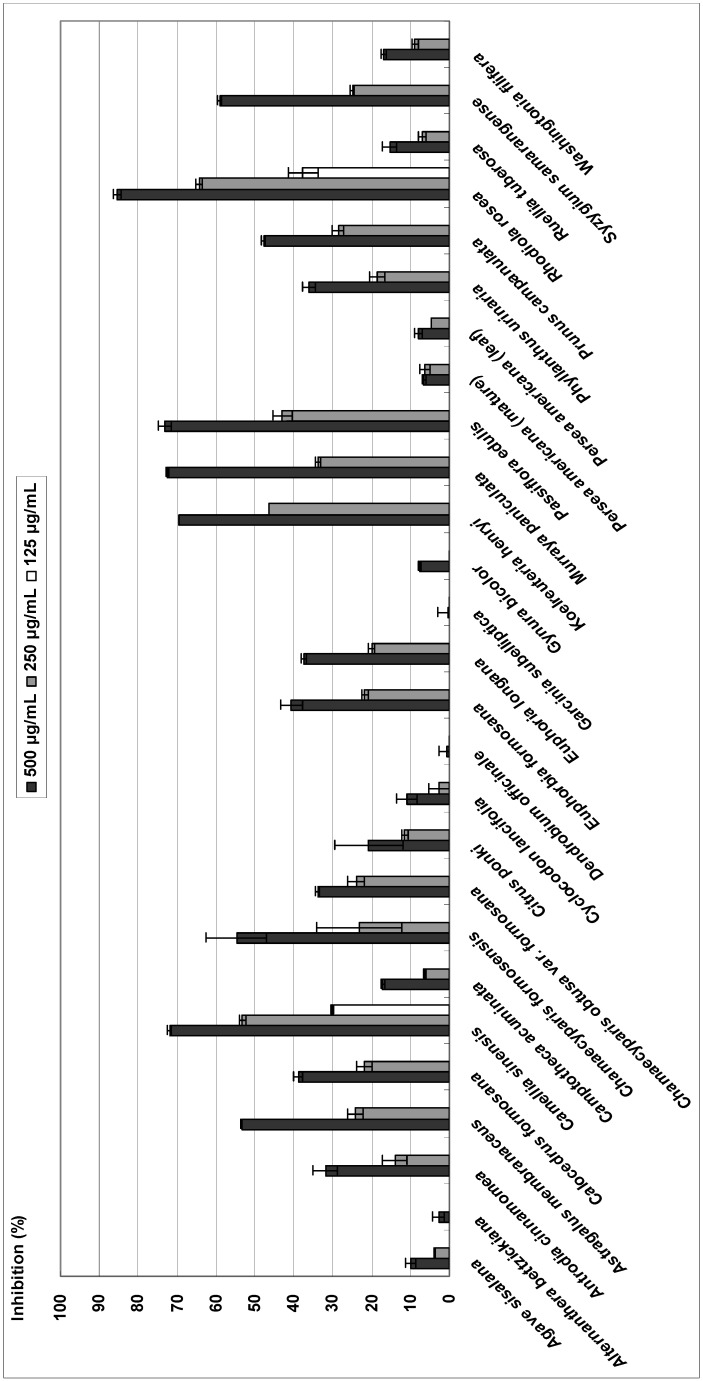
Inhibitory effect of 27 plant acetone extracts on tyrosinase activity.

All plant extracts were screened for tyrosinase inhibitory activity. The assay was carried out at three different extract concentrations: 125, 250 and 500 μg/mL. Of the extracts assayed, 27 demonstrated tyrosinase inhibitory activity at 500 μg/mL, among which eight (29.9%) had inhibition rates >50%. Two extracts (7.8%) were found to be active at a concentration of 250 μg/mL and exhibited inhibition >50%. At 125 μg/mL, no extracts were active or exhibited inhibition >50%. The crude extracts possessing tyrosinase inhibitory activity with IC_50_ values less than 300 μg/mL were acetone extracts of *Koelreuteria henryi* (IC_50_, 289.0 ± 0.5 μg/mL), *Camellia sinensis* (IC_50_, 232.5 ± 3.3 μg/mL) and *Rhodiola rosea* (IC_50_, 181.8 ± 11.0 μg/mL). In comparison, the IC_50_ for kojic acid, which was used as a postivie control for inhibition of tyrosinase, was 6.2 ± 0.4 μg/mL (Figure 2). *Camellia sinensis* has previously been reported to inhibit tyrosinase [12]. Here we report the first measurement of the tyrosinase inhibitory activities of *Koelreuteria henryi* and *Camellia sinensis*. Extracts from these plants may be a potential source of new inhibitors towards tyrosinase.

### Lipoxygenase

**Figure 3:**
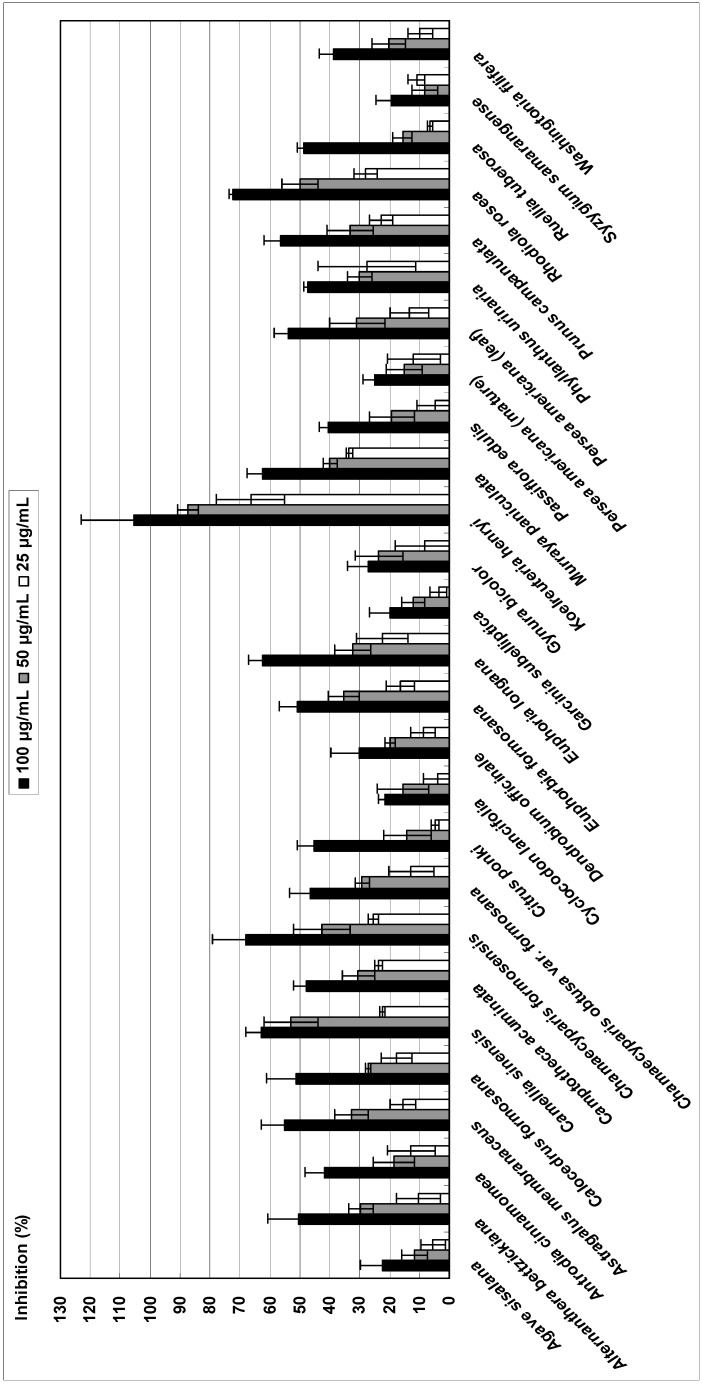
Inhibitory effect of 27 plant acetone extracts on lipoxygenase activity.

The LOX inhibitory activity of 27 crude extracts at concentrations of 25, 50, 100 μg/mL was investigated and compared to that of quercetin (Figure 3). Of the extracts assayed, 27 extracts demonstrated LOX inhibitory activity at 100 μg/mL, among which nine (33.3%) showed an inhibition rate >50%. Three extracts (11.1%) were found to be active at a concentration of 50 μg/mL, among which two (7.8%) exhibited inhibition >50%. At 25 μg/mL, only one extract had an inhibition rate >50%. The crude extracts possessing LOX inhibitory activity with IC_50_ values <50 μg/mL were acetone extracts of *Koelreuteria henryi* (IC_50_, 17.5 ± 4.3 μg/mL), *Camellia sinensis* (IC_50_, 49.1 ± 7.6 μg/mL) and *Rhodiola rosea* (IC_50_, 49.6 ± 13.6 μg/mL). The IC_50_ value for quercetin was 8.6 ± 0.8 μg/mL(28.5 μM). The extract from *Koelreuteria henryi* exhibited excellent inhibition of LOX activity. The high antioxidant capacity of *Koelreuteria henryi* may be due to the presence of flavonoids or polyphenols in the extract. LOX activities pertaining to *Koelreuteria henryi*, *Camellia sinensis* and *Rhodiola rosea* have not previously been reported. Further study of these plants is warranted to identify and isolate the compound(s) responsible for LOX inhibition.

### HPLC-DAD for Koelreuteria henryi with tyrosinase

Plants contain a mixture of many kinds of secondary metabolism products, including phenolics, which vary greatly in their antioxidant capacity. A method is needed to rapidly identify which components are the most active and contribute the greatest to the plant's antioxidant capability. Our analysis began with a rapid spectrophotometric screening of all plant extracts to determine which had the highest antioxidant activity via inhibition assays for XO, tyrosinase and LOX. Following this, the candidates with highest activity were chosen for tyrosinase and HPLC-DAD analysis to validate the antioxidant action of any botanical constituents that demonstrated activity. HPLC-DAD can detect a powerful radical scavenger from a complex mixture, with the real time data showing changes, as antioxidant peaks disappear or change to other peaks. Further structural characterization can then be implemented to learn more about the active constituents [15]. Therefore, the powerful antioxidant constituent would be the corresponding decreased or lost peak on the HPLC-DAD chromatogram.

Under the appropriate reaction conditions, a more active compound is displayed as a faster reducing peak owing to its reactivity, which may enable one to screen the most active compound in the raw extract by using a simple HPLC-DAD instrument. Here we report the use of this method with tyrosinase as an enzyme species for the screening of the powerful antioxidant compounds in *Koelreuteria henryi* extract.

**Figure 4:**
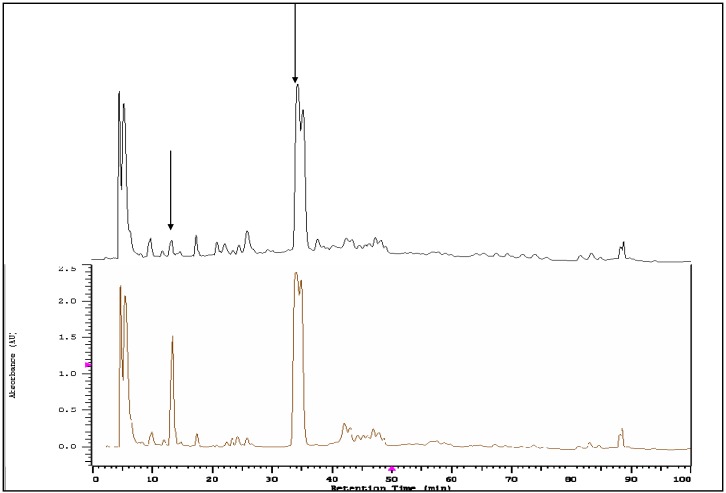
Of the 27 plant extracts that were screened, only the extract from *Koelreuteria henryi* exhibited strong xanthine oxidase, tyrosinase, and lipoxygenase inhibitory activity thus indicating this extract was an appropriate candidate for HPLC-DAD analysis. Upper line: HPLC-DAD chromatogram of *Koelreuteria henryi* extract with tyrosinase. Lower line: HPLC-DAD chromatogram of *Koelreuteria henryi* extract without tyrosinase.

Figure 4 shows the gradient HPLC-DAD chromatogram of the total constituents of *Koelreuteria henryi* extract with tyrosinase (represented as a black line) in comparison with no tyrosinase (represented as a brown line). The classical fractionation procedure of *Koelreuteria henryi* without the addition of the tyrosinase is very time-consuming for identifying antioxidant compounds from the extract, and may produce an undesirable result due to decomposition of the antioxidant compounds during the repeated fractionation. During the HPLC-DAD assay where tyrosinase is added, the separation and detection of the antioxidant are completed at the same time.

The intensities of the peaks at 13.49 and 35.28 minutes (Figure 4) were obviously decreased by the addition of tyrosinase when compared to those in the absence of tyrosinase. The peaks marked with an arrow indicate compounds that most likely contribute to the strong anti-tyrosinase activity of the *Koelreuteria henryi* extract. Of all the Taiwanese plant extracts that were screened, only the extract from *Koelreuteria henryi* exhibited strong XO, tyrosinase, and LOX inhibitory effects. It would be of interest to further isolate and elucidate the structures of the XO, tyrosinase, and LOX inhibitors in the extract.

## Experimental

### General

For reverse phase chromatography, A HPLC-DAD (Hitachi L-7110) instrument equipped with a diode array detector (Hitachi L-7455), and reverse phase column (BioSil, Aqu-ODS-W5*u*, 4.6 × 250 mm, flow rate 1 mL・min^-1^) were used to analyze the compounds. All chemicals and enzyme were purchased from Merck and Sigma Company.

### Plant material

All plant materials were chosen and collected from the Puli Barch Station, Taichung District Agricultural Research and Extension Station, Council of Agriculture. The voucher specimens of plants were deposited in the College of Pharmacy, Taipei Medical University, Taiwan.

### Extraction and isolation

Plants were pulverized and subsequently extracted with acetone [16]. Acetone extraction was chosen over ethanol, methanol, chloroform and other solvents because it has lower toxicity [17,18] The extracts were concentrated *in vacuo*, freeze-dried and stored at −20 °C until used (Table 1). The physiological actions of the plant acetone extracts were determined by measuring the IC_50_ values as Cui *et al*. [19]

### Xanthine oxidase inhibition assay

The XO inhibitory activity was assayed spectrophotometrically under aerobic conditions, based on the procedure reported by Noro *et al*. [20]. The assay mixture, consisting of 50 μL of test solution, 35 μL of 0.1 mM phosphate buffer (pH=7.5), and 30 μL of enzyme solution (0.01 units/ml in 0.1 mM phosphate buffer, pH=7.5), was prepared immediately before use. After preincubation at 25°C for 15 minutes, the reaction was initiated by the addition of 60 μL of substrate solution (150 mM xanthine in the same buffer). The assay mixture was incubated at 25 °C for 30 minutes. Absorbance at 290 nm was measured with a Thermo Varioskan Flash spectrophotometer (Thermo Electron Corp, Vantaa, Finland). A blank was prepared in the same manner. One unit of XO was defined as the amount of enzyme required to produce 1 mmol of uric acid/minute at 25 °C. XO inhibitory activity is expressed as the percentage inhibition of XO in the above system, calculated as (1-*B*/*A*) × 100, where *A* and *B* are the activities of the enzyme without and with test material. IC_50_ values were calculated from the mean values of data from three determinations. The crude extracts were dissolved initially in DMSO, followed by dilution with the buffer; the final concentration of DMSO was less than 0.25%. Quercetin, a known inhibitor of XO, was used as a positive control.

### Tyrosinase inhibition assay

The tyrosinase inhibitory activity was measured as previously described, using tyrosine as the substrate [21]. Mushroom tyrosinase aqueous solution (40 μL, 50 IU/mL), phosphate buffer (pH 6.8) (160 μL) (*A*), or phosphate buffer (pH 6.8, 120 μL) along with test samples (40 μL) (*B*) were mixed. Extracts were tested at a concentration of 500 μg/ml to assess the inhibitory effect on tyrosinase in vitro. Kojic acid (0.1 mg/mL in DMSO) was used as positive control. The mixture was preincubated at 37 °C for 5 minutes, and then L-tyrosine (80 μL, 0.1 mg/mL) was added. The mixture was then incubated for 30 minutes at 37°C. The amount of dopachrome was measured at 475 nm using a microplate spectrophotometer (Molecular Devices SpectraMAX 340PC, Teken Scientific Corp.). The IC_50_ value was obtained by extrapolation from linear regression analysis and denoted the concentration of sample required to inhibit 50% of tyrosinase activity. The percent inhibition of tyrosinase activity was calculated as follows: % inhibition = (A – B)/A × 100. Where A=absorbance at 475 nm without a test sample, and B=absorbance at 475 nm with a test sample. 

### Lipoxygenase inhibition assay

The lipoxygenase activity was measured as described previously [22] in borate buffer (0.2 M, pH 9.00) at 234 nm after addition of 15-LO, using linoleic acid (134 μM) as a substrate. The final enzyme concentration was 167 U/mL. Test substances were added as DMSO solutions (final DMSO concentration of 1.6%). The enzyme solution was stored on ice, and controls were measured at intervals throughout the experimental period to ensure that enzyme activity was constant. Quercetin, a well-known inhibitor of 15-LOX, was employed as a positive control. The IC_50_ values were determined by linear interpolation by the measuring points closest to 50% activity.

The absorbance at 234 nm was measured with a Thermo Varioskan Flash spectrophotometer (Thermo Electron Corp.). The percent inhibition of LOX activity was calculated as follows: % inhibition = (A–B)/A×100, where A=absorbance at 234 nm without a test sample, and B = absorbance at 234 nm with a test sample.

### Detection of antioxidant compounds in a plant extract of Koelreuteria henryi by HPLC-DAD

The *Koelreuteria henryi* (15 mg) acetone extract was dissolved in DMSO (75 μL) and buffer solution (525 μL, 50 mM CH_3_COONa, pH 6) and CH_3_CN (900 μL) were added to obtain a 10 mg/mL blank solution. Similarly, the *Koelreuteria henryi* (15 mg) acetone extract was dissolved in DMSO (75 μL), buffer solution (425 μL, 50 mM CH_3_COONa, pH 6) and CH_3_CN (900 μL) and tyrosinase (100 μL, 716 units/mL) were added to obtain a 10 mg/mL test solution. Both mixtures were well stirred and allowed to stand for 40 minutes at 38°C. The solution (10 μL) was injected into the HPLC-DAD and analyzed under the following conditions: column, Biosil ODS-W 5u 4.6 × 250 mm; solvent A, HFBA (pH 2.8); solvent B, CH3OH; gradient systems, a linear gradient of solvent A from 100% to 80% for 10 minutes, from 80% to 50% for 70 minutes, from 40% to 0% for 10 minutes, and then 100% of solvent B (10 minutes) for the extracts from *Koelreuteria henryi*; flow rate, 1 mL/minute; detection, 218 nm; detector, Diode array detector Hitachi L-7455.

### Statistical analysis

Data are presented as the mean ± standard deviation (S.D.) of each triplicate test.
